# Changes in bone mineral density after total parathyroidectomy without autotransplantation in the end-stage renal disease patients with secondary hyperparathyroidism

**DOI:** 10.1186/s12882-018-0934-1

**Published:** 2018-06-15

**Authors:** Li Fang, Jining Wu, Jing Luo, Ping Wen, Mingxia Xiong, Jinlong Cao, Xiaolan Chen, Junwei Yang

**Affiliations:** 1 Department of Nephrology, Affiliated Hospital of Nantong University, 20 Xisi Road Nantong, Nantong, Jiangsu Province China; 2 Department of Nephrology, Second Affiliated Hospital of Nanjing Medical University, 262 Zhongshan North Road, Nanjing, Jiangsu Province China

**Keywords:** Bone mineral density, Total parathyroidectomy without autotransplantation, Secondary hyperparathyroidism, Dual-energy X-ray absorptiometry, End-stage renal disease

## Abstract

**Background:**

The patients with secondary hyperparathyroidism (SHPT) usually had reduced bone mineral density, which might lead to a substantial increase in osteoporosis, fracture and mortality. Although surgical intervention is effective in reducing parathyroid hormone (PTH) levels in suitable candidates refractory to medical therapy, the effect of surgery on bone mass changes still requires further evaluation. Thus, the aim of this study was to evaluate the characteristics of BMD changes after total parathyroidectomy (PTX) without autotransplantation and its associated factors.

**Methods:**

The records of 34 patients who underwent successful total PTX without autotransplantation with a preoperative and postoperative dual energy X-ray absorptiometry (DEXA) scan in our institution within 4 years of operative intervention were reviewed. Correlation and regression analysis were used to identify factors that independently predict BMD changes.

**Results:**

At baseline, we found that the prevalence of osteoporosis seemed to be much higher in the load-bearing lumbar spine than in the hip, varying greatly even between different lumbar vertebrae. The bone loss in SHPT had its predilection site in the load-bearing cancellous bone. After curative total PTX without autotransplantation, BMD improved significantly in both lumbar spine and hip overall. The largest increase in BMD occurred at L4 vertebrae with the lowest pre-operative BMD. At the most affected site L4, BMD improved in up to 94.1% of patients: 86.2% had significant improvement, 5.9% moderate improvement, and 5.9% declining bone mineral density. Correlation and regression analysis suggested that percentage changes in BMD were predicted negatively by the preoperative BMD and positively by the preoperative parathyroid mass but not intact PTH levels.

**Conclusion:**

Total parathyroidectomy without autotransplantation could improve BMD of secondary hyperparathyroidism at L1-L4 and the hip. Furthermore, the large parathyroid glandular mass and the preoperative BMD predicted the BMD changes after surgery.

**Electronic supplementary material:**

The online version of this article (10.1186/s12882-018-0934-1) contains supplementary material, which is available to authorized users.

## Background

Renal osteodystrophy is one of the major serious complications in patients with end stage renal disease (ESRD) receiving long-term hemodialysis [1]. In addition to the abnormalities of bone architecture that contribute to fracture risk [2, 3], renal osteodystrophy is also associated with the development of arterial calcification and cardiovascular disease [4–6]. Increasing risks of fractures, together with high cardiovascular morbidity and mortality rates, have recently drawn much attention to devise an effective treatment plan for renal osteodystrophy that hopefully will lead to an improved outcome.

Osteitis fibrosa cystica, which is the most commonly encountered bone abnormality in renal osteodystrophy, classically appeared as an increased bone turnover that was not only associated with reduced bone density but also with osteopenia and osteoporosis that predisposes to an increased risk of fracture [7]. Since secondary hyperparathyroidism (SHPT) characterized by persistent elevated parathyroid hormone (PTH) and marked parathyroid hyperplasia is the leading cause of osteitis fibrosa cystica [8], the current recommended treatment for renal osteodystrophy is to manage secondary hyperparathyroidism, but not typical osteoporosis therapies. At the early stage, improvement of renal osteodystrophy would be an expected consequence of effectively suppressing PTH with phosphate binders, calcitriol analogs and calcimimetics. Whereas, at the late stage, the progressively reducing expression of calcium-sensing receptors and vitamin D receptors might result in resistance to medical therapy and surgical intervention might be gradually necessary to achieve adequate control of refractory secondary hyperparathyroidism [9–14]. However, it has also been reported that very low PTH levels and over-suppression of PTH were linked with adynamic bone disease, decreased bone mineral density (BMD) and a greater risk for fracture among long-term dialysis patients [15–17]. The impact of surgical intervention on bone health in secondary hyperparathyroidism remains uncertain, especially total parathyroidectomy without autotransplantation.

Thus, in this study, we sought to investigate the effect of total parathyroidectomy without autotransplantation on bone mineral density in the end-stage renal disease patients with refractory secondary hyperparathyroidism. Besides, we also examined the preoperative and postoperative clinical parameters to identify predictive factors related to the postoperative BMD changes.

## Methods

### Participants

We performed a retrospective analysis of 712 patients, who underwent total parathyroidectomy (PTX) without autotransplantation treatment for refractory secondary hyperparathyroidism in the second affiliated hospital of Nanjing Medical University between January 2009 and December, 2015. Refractory SHPT was defined as following: 1) After regular drug treatment, serum intact PTH level > 500 pg/mL on two or more occasions; and/or 2) Patients with parathyroid nodular or diffuse hyperplasia identified by ultrasound imaging or radioisotope scan; 3) Patients with symptomatic secondary hyperparathyroidism, such as bone and joint pain, pathologic fractures, severe pruritus, restless legs syndrome and so on. All operations were carried out by the same surgical team. We identified 34 of these patients with a preoperative and postoperative DEXA scan in our institution within 4 years of operative intervention. Patients were excluded if either their pre- or postoperative DEXA scan was performed at an outside institution, if they were not cured from surgery (The relative reduction of 24 h-postoperative iPTH< 90%), or if they declined participation in research studies. The baseline characteristics of age, sex, duration of dialysis between the study participants and those excluded have no significant difference, as shown in Additional file 1: Table S1. However, the preoperative PTH levels were much higher in those excluded. In all patients, 4 h hemodialysis was performed three times a week using TORAY TR8000 machines. High flux polysulfone membrane (T-sulfone, Toray, Japan) were used. For dialysis of normal-size adults, the blood flow rate is usually set between 250 and 300 mL/min. Individual ultrafiltration rates were held constant, from 500 ml/h to 800 ml/h to achieve dry weight.

All procedures performed in the study involving human participants were in accordance with the ethical standards of the institutional and/or national research committee and with the 1964 Helsinki declaration and its later amendments or comparable ethical standards. Besides, this study was also approved by the ethics committee of Nanjing Medical University. There was no commercial sponsorship.

### Blood measurements

Blood samples were obtained before the start of the hemodialysis session in a 12 h-fasting state. Blood routine tests were performed using an LH-750 Hematology Analyzer (Beckman Coulter, Inc., Fullerton, CA). Laboratory assessment included blood urea nitrogen, serum creatinine, serum albumin, high-density lipoprotein cholesterol (HDLC), low-density lipoprotein cholesterol (LDLC), total cholesterol, triglyceride, magnesium, calcium, and phosphorus were performed using photometric method by Automatic Biochemical Analyzer (HITACHI 7080; Hitachi, Ltd., Tokyo, Japan). Serum iPTH levels were measured using a UniCel DxI800 Access Immunoassay System (Beckman Coulter, Inc., Fullerton, CA).

### BMD measurements

Bone mineral density (BMD) were performed on Hologic Discovery duel-energy x-ray absorptiometry scanner, using the manufacturer’s recommended standard procedures for the postero-anterior lumbar spine at L1–L4, and the proximal femur at the femoral neck, trochanter, intertrochanteric region and total region. Standard deviation from the mean value of younger healthy ones was expressed as T-score, while the standard deviation from the mean value of age and gender-matched controls was expressed as Z-score. The results were evaluated according to World Health Organization (WHO) osteoporosis criteria: (a) normal, a BMD T-score greater than − 1 SD; (b) osteopenia, a T-score between − 1 and − 2.5 SD; (c) osteoporosis, a T-score below − 2.5 SD.

### Statistical analyzes

Statistical analysis was done with SPSS 15 J for Windows (SPSS, Chicago, IL, USA) to observe any significant differences. Data were presented as the mean (SD) for continuous variables with a normal distribution or median (interquartile range) for those with a skewed distribution. Student’s paired t test was used to compare preoperative BMD values and biochemical values with those obtained after surgery. To investigate the possible variables influencing BMD changes, both univariate Spearman’s correlation and stepwise multivariate linear regression analysis are used. We first used the univariate Spearman’s correlation to analysis the relationship between BMD changes and the clinical parameters. When the variables are highly correlated, stepwise regression models in which the choice of predictive variables is carried out by an automatic procedure were then used to choose the independent variables. Differences were considered significant when *P* value was less than 0.05.

## Results

Baseline demographics, clinical characteristics and biochemical profiles of our participants were presented in Table 1**.** The study included 34 patients (14 women and 20 men) with a mean age of 49.7 ± 8.44 years (range, 31–65 years). The mean duration of dialysis before surgery was 84 (60–144) months. The serum intact PTH concentration (median and inter-quartile range) was 852.0 (637.50–1058.78) pg/ml. Besides, the derangements of calcium/phosphate homeostasis with serum phosphorus 2.23 ± 0.409 mmol/L and serum calcium 2.56 ± 0.211 mmol/L were also common seen.

**Table 1:** Baseline characteristics of the subjects with secondary hyperparathyroidism

	Patients (*n* = 34)
Age, years	49.7 ± 8.44
Gender (male/female)	20/14
Dialysis vintage, months	84 (60–144)
Kt/V	1.4 ± 0.19
Pre-PTX BMI	23.6 ± 4.91
Systolic blood pressure, mmHg	134.4 ± 16.34
Diastolic blood pressure, mmHg	77.8 ± 15.27
Hemoglobin, g/L	114.6 ± 18.83
C-reactive protein, mg/L	14.6 ± 29.78
Serum creatinine, umol/L	952.2 ± 240.79
Albumin, g/L	44.8 ± 3.87
Alkaline phosphatase, U/L	165.9 ± 115.81
Calcium, mg/dl	2.56 ± 0.211
Phosphorus, mg/dl	2.23 ± 0.409
Pre-PTX intact PTH, pg/ml	852.0 (637.5–1058.78)
Post-PTX intact PTH, pg/ml	1.2 (0.8–2.5)

Continuous data are presented as mean ± SD when normally distributed and as median (interquartile range) when skewed

To evaluate the effect of total parathyroidectomy without autotransplantation on BMD changes, we firstly assessed the characteristics of regional bone mineral density at lumbar spine and hip by dual energy X-ray absorptiometry before surgery. We found that there were significant differences in BMD values between various anatomic bone sites as shown in Additional file 2: Table S2. According to the WHO criteria based on T-scores, for lumbar spine, osteoporosis at L1 was revealed in 11.8% patients while at L4 was in 32.4% patients; for the hip, the femoral neck yielded the highest frequency of diagnosis of osteoporosis (23.5%) and next came the intertrochanter (17.6%). These data indicated that the bone involvement in severe secondary hyperparathyroidism had its predilection site and the assessment of changes should be taken separately for different anatomical sites.

**Fig. 1:**
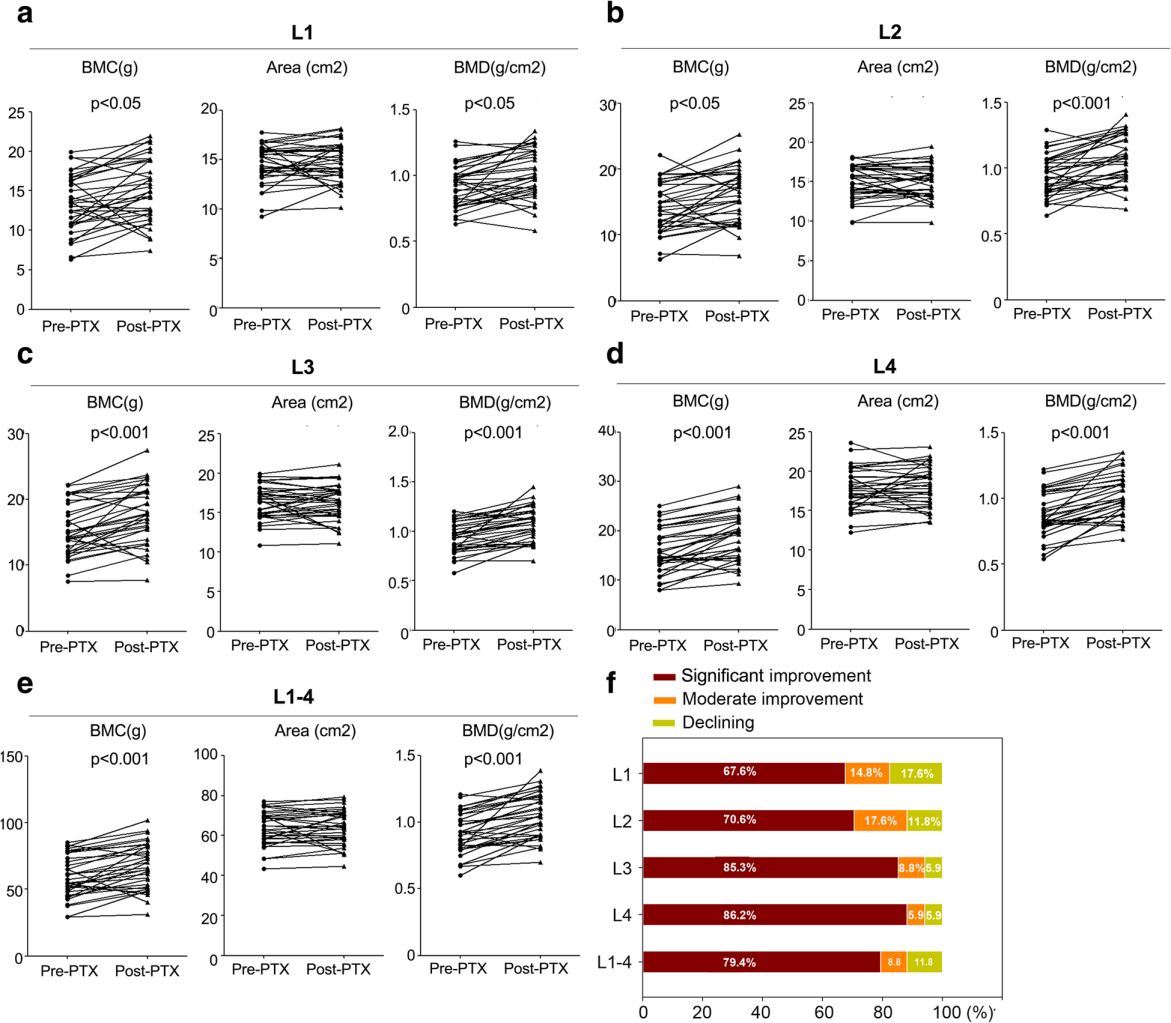
Before and after surgery, the changes of bone mineral content (BMC), bone area and BMD in the lumbar spine region. **a**. The changes of BMC, bone area and BMD at L1; **b**. The changes of BMC, bone area and BMD at L2; **c**. The changes of BMC, bone area and BMD at L3; **d**. The changes of BMC, bone area and BMD at L4; **e**. The changes of BMC, bone area and BMD at L1–4; **f**. Distribution of patients with changes in bone mineral density compared to preoperative T-score through separate analyses. *, *P* value of Student’s paired t test between pre- and post-PTX values < 0.05

**Fig. 2:**
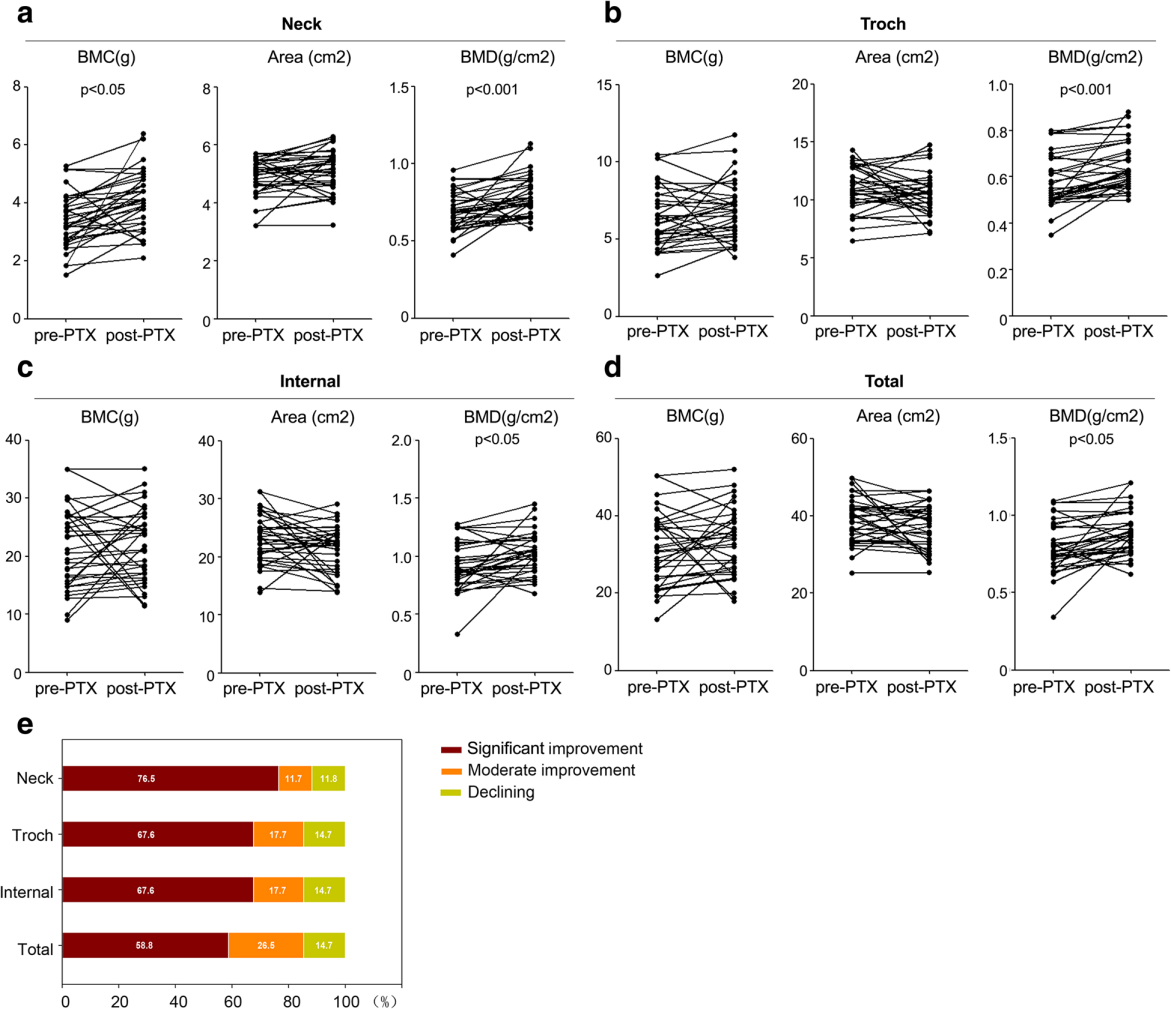
Before and after surgery, the changes of BMC, bone area and BMD in the hip region. **a**. The changes of BMC, bone area and BMD at femoral neck; **b**. The changes of BMC, bone area and BMD at trochanter; **c**. The changes of BMC, bone area and BMD at intertrochanteric region; **d**. The changes of BMC, bone area and BMD in the hip. **e**. Distribution of patients with changes in bone mineral density compared to preoperative T-score through separate analyses. *, *P* value of Student’s paired t test between pre- and post-PTX values < 0.05

Overall, as presented in Additional file 2: Table S2, bone mineral density had improved significantly after total parathyroidectomy. The increases in BMD and T-scores were much greater for the lumbar spine than for the hip. When we analyzed the characterization of improvement in details, in the lumbar area, we found that the increase in bone mineral content was significant while the change in bone area was not obvious (as shown in Fig. 1a-e); while in the hip, although not statistically significant, we found the increase in bone mineral content was always accompanied by the reduction of bone area (as shown in Fig. 2a-d). According to the previous research [18], we likewise defined significant improvement if the patients’ BMD increased > 5% during the postoperative follow-up period and moderate improvement if they had a BMD increase of 0.1–5%. As illustrated in Figs. 1f and 2e, we found that the largest increases in BMD occurred at sites with the lowest pre-operative BMD. At the most frequently affected sites in the lumbar region L4, 86.2% of patients had significant improvement (> 5%) in BMD, 5.9% had moderate improvement (0.1–5%) and 5.9% had declining bone mineral density. Similarly, in the hip region, 76.5% of patients had significant improvement at the femoral neck where yielded the highest frequency of diagnosis of osteoporosis.

On correlation analysis (as shown in Table 2), we found that the percentage change in L1-L3 vertebra BMD but not hip were positively correlated with preoperative intact PTH, postoperative changes of PTH in 24 h and mass of preoperative parathyroid glands. Yet no correlations were observed between BMD changing rates with ages, gender, dialysis vintage, weight and height and time after surgery in both the lumbar spine and hip. As illustrated in Additional file 3**:** Figure S1**,** when we divided the patients into three groups according to the postoperative durations, we found that the postoperative durations did not affect the changes in BMD after surgery. Instead, there was a trend of correlation between percentage change in BMD and the preoperative bone lesions. In the multiple linear regression analysis **(**Table 3**)**, we unexpectedly found that the BMD changes in most measured regions were affected by the mass of resected parathyroid glands. Besides, preoperative BMD, preoperative height, preoperative weight also participated in explaining the changes of BMD.

**Table 2:** Univariate Spearman’s correlation coefficients for the relationships between clinical parameters and percentage change in BMD at the lumbar spine and hip after total parathyroidectomy (PTX) without autotransplantation

		Percentage change in BMD at the LS				Percentage change in BMD at the hip		
		L1	L2	L3	L4	Neck	Troch	Internal
Age (years)	r	0.143	− 0.055	− 0.149	− 0.183	− 0.142	− 0.183	− 0.100
	p	0.418	0.758	0.401	0.300	0.422	0.301	0.575
Gender	r	−0.061	− 0.055	− 0.018	− 0.079	0.061	− 0.006	0.055
	p	0.732	0.758	0.918	0.656	0.732	0.973	0.758
Postoperative period(years)	r	0.131	0.062	0.106	0.161	0.279	−0.048	0.044
	p	0.461	0.727	0.552	0.362	0.111	0.788	0.804
Dialysis vintage(months)	r	0.028	−0.031	−0.163	−0.046	− 0.059	−0.052	− 0.127
	p	0.874	0.860	0.357	0.797	0.742	0.769	0.474
Pre-PTX intact PTH	r	**0.363** ^*^	**0.367** ^*^	**0.427** ^*^	0.153	0.061	0.280	−0.083
	p	**0.035**	**0.033**	**0.012**	0.387	0.733	0.108	0.639
Post-PTX Changes of PTH in 24 h	r	**0.377** ^*^	**0.382** ^*^	**0.446** ^**^	0.172	0.072	0.300	−0.071
	p	**0.028**	**0.026**	**0.008**	0.330	0.685	0.085	0.690
Weight of resected parathyroid glands	r	**0.366** ^*^	**0.327**	**0.452** ^**^	0.267	0.097	0.142	−0.067
	p	**0.033**	**0.059**	**0.007**	0.126	0.584	0.424	0.705
Mass of resected parathyroid glands	r	0.326	0.287	**0.374** ^*^	0.198	0.044	0.135	−0.087
	p	0.060	0.100	**0.029**	0.261	0.805	0.446	0.624
Pre-PTX bone area	r	−0.102	−0.090	− 0.226	− 0.214	**− 0.477** ^**^	**− 0.381** ^*^	**−0.392** ^*^
	p	0.567	0.613	0.198	0.224	**0.004**	**0.026**	**0.022**
Pre-PTX BMC	r	−0.305	−0.226	**−0.355** ^*^	**− 0.375** ^*^	**−0.561** ^**^	**− 0.557** ^**^	**−0.618** ^**^
	p	0.080	0.200	**0.039**	**0.029**	**0.001**	**0.001**	**0.000**
Pre-PTX BMD	r	**−0.339** ^*^	−0.292	**− 0.372** ^*^	**− 0.392** ^*^	**− 0.555** ^**^	**− 0.596** ^**^	**−0.594** ^**^
	p	**0.050**	0.094	**0.030**	**0.022**	**0.001**	**0.000**	**0.000**
Pre-PTX T-score	r	**−0.354** ^*^	−0.282	**− 0.357** ^*^	**− 0.372** ^*^	**−0.506** ^**^	**− 0.549** ^**^	**− 0.491** ^**^
	p	**0.040**	0.106	**0.038**	**0.030**	**0.002**	**0.001**	**0.003**
Pre-PTX Z-score	r	**−0.403** ^*^	−0.351	−0.341	− 0.325	**−0.543** ^**^	**− 0.580** ^**^	**−0.518** ^**^
	p	**0.027**	0.057	0.065	0.080	**0.002**	**0.001**	**0.003**

* Significant at *p* < 0.05 level; ** Significant at *p* < 0.01 level. *p* values in bold indicate significance levels

**Table 3:** Stepwise multivariate linear regression models of changes in BMD for the lumbar spine and hip

	β	SE	*p* value
Lumbar Spine			
Percentage change in BMD at L1			
Mass of resected parathyroid glands	0.021	0.003	0.000
Pre-PTX height	0.005	0.002	0.033
R2 for the model = 0.648			
Percentage change in BMD at L2			
Mass of resected parathyroid glands	0.019	0.003	0.000
Pre-PTX BMD	−0.420	0.153	0.012
Pre-PTX height	0.005	0.002	0.030
R2 for the model = 0.692			
Percentage change in BMD at L3			
Mass of resected parathyroid glands	0.019	0.003	0.000
Pre-PTX BMD	−0.389	0.127	0.005
R2 for the model = 0.746			
Percentage change in BMD at L4			
Mass of resected parathyroid glands	0.015	0.003	0.000
Pre-PTX BMD	−0.498	0.137	0.001
Pre-PTX Albumin	0.011	0.005	0.034
R2 for the model = 0.654			
Hip			
Percentage change in BMD at femoral neck			
Pre-PTX BMC	−0.166	0.039	0.000
Mass of resected parathyroid glands	0.017	0.005	0.003
R2 for the model = 0.618			
Percentage change in BMD at trochanter			
Mass of resected parathyroid glands	0.018	0.003	0.000
Pre-PTX BMC	−0.053	0.010	0.000
R2 for the model = 0.765			
Percentage change in BMD at intertrochanter			
Pre-PTX weight	0.010	0.002	0.000
Pre-PTX BMD	−0.658	0.125	0.000
R2 for the model = 0.769			

*Significant at *p* < 0.05 level; ** Significant at *p* < 0.001 level

## Discussion

Secondary hyperparathyroidism, which was ubiquitous in patients on maintenance hemodialysis, has now been recognized as the predominant cause of renal osteodystrophy. In the early stage, by targeting secondary hyperparathyroidism, renal osteodystrophy could easily be managed with medical treatment. However, in the late stage, despite considerable advances in medical therapies, surgical treatment which has already proven to be much more cost effective in the long term remains necessary for treating refractory disease at times [13]. Yet surgical consensus on the optimal procedure has not been reached, total parathyroidectomy without autotransplantation is gradually accepted and recommended because of the higher cure rate and the lower recurrence rate [14, 19–22]. Concerning the possible adverse impact on bone health in absence of PTH, our present study herein was mainly focused on the bone mineral status in secondary hyperparathyroidism and the effect of total parathyroidectomy without autotransplantation on bone density changes.

In concordance with previous studies, our data demonstrated that most of the patients with refractory SHPT displayed a marked reduction of bone density. And the BMD reduction was preferentially seen at cancellous (e.g. lumbar spine) versus cortical bone sites, although protracted elevation of serum parathyroid hormone (PTH) was held to be associated with cortical, but not cancellous, bone loss [23–25]. After successful total parathyroidectomy without autotransplantation, further bone loss could be halted and BMD subsequently increased at nearly all skeletal sites. The lumbar spine showed a greater increase in BMD than the hip and the greatest increase in BMD was observed in the weight-bearing L4. This indicated that the sites with lowest BMD might benefit most from surgical cure. However, unlike the increase observed by Mohamed et al. in their parathyroidectomized patients [26], we observed the most marked regain in areas rich in cancellous bone whereas they found the largest increase at sites with predominantly cortical bone. Although the mechanisms underlying this discrepancy is not evident, but in both studies the largest increase in bone density occurred at the sites with the lowest preoperative BMD.

In addition to preoperative bone abnormalities, the effect of curative parathyroid resection on BMD changes was also affected by the preoperative hyperparathyroidism status, but not by age, gender, dialysis vintage and postoperative period. In the previous studies, Mohamed et al. have demonstrated that the parathyroidectomy resulted in an increased bone mass during the first 6–12 months after surgery [26]; Aiji Yajima et al. have further demonstrated that the osteoblast number increased 1 week after surgery compared with before parathyroidectomy, and the production of lamellar osteoid was detected [24]. These studies suggested that the postoperative BMD might increase mainly within the first year. That might be the reason why we found that the postoperative durations did not affect the changes in BMD after surgery. Most interestingly, with Spearman’s correlation, we found that percentage changes in BMD were correlated with both preoperative intact PTH levels and preoperative parathyroid masses; whereas, with linear regression which was determined as the best way to predict effect from cause, percentage changes in BMD were predicted only by preoperative parathyroid masses but not intact PTH levels. As we pointed in the previous study [9], PTH levels might be influenced by multiple pharmaceutical drugs in short term, while the size of parathyroid gland might be a much more accurate marker reflecting the long-term hyperparathyroidism status. Base on this point, our data suggested that the bone mineral status in secondary hyperparathyroidism and changes after surgical cure were strongly associated with chronic hyperparathyroidism status. However, since our study was based on the retrospective and uncontrolled data, there are many limitations of our study, such as selection bias, lack of close follow up, lack of data on bone biopsy, bone metabolic markers, and fracture risk. In the future, well designed randomized controlled trials are still needed to come to a solid conclusion.

## Conclusion

In summary, despite the limitation of a small sample size, the lack of matched control group and the lack of bone histomorphometric data, our present study, in combination with previous report [27, 28], still provided some useful bone informations that amelioration of biochemical consequences of secondary hyperparathyroidism by total parathyroidectomy without autotransplantation might have a positive effect in BMD.

## Additional files

Additional file 1: **Table S1.** The baseline characteristics of the study participants and those excluded. (DOC 27 kb)

Additional file 2: **Table S2.** Results of dual X-ray absorbtiometry (DXA) before and after total parathyroidectomy (PTX). (DOC 51 kb)

Additional file 3: **Figure S1.** BMD changes after surgery in the participants with different postoperative durations. A-E: BMD changes in the lumbar spine region; G-I: BMD changes in the hip region. (TIF 37610 kb)

## Abbreviations

BMC: Bone mineral content
BMD: Bone mineral density
DEXA: Dual energy X-ray absorptiometry
ESRD: End-stage renal disease
HDLC: High-density lipoprotein cholesterol
i-PTH: Intact-parathyroid hormone
LDLC: Low-density lipoprotein cholesterol
PTX: Parathyroidectomy
SHPT: Secondary hyperparathyroidism
WHO: World Health Organization
