# Supplementary material for: Changes in bone mineral density after total parathyroidectomy without autotransplantation in the end-stage renal disease patients with secondary hyperparathyroidism
Source: BMC Nephrol. 2018 Jun 15;19:142. doi: 10.1186/s12882-018-0934-1 (PMC6003160; doi:10.1186/s12882-018-0934-1)
Supplement: Supplementary file 1 — Table S1. The baseline characteristics of the study participants and those excluded. (DOC 27 kb) [file 12882_2018_934_MOESM1_ESM.doc]

|  | Age | Sex (male/female) | Duration of dialysis,months | Preoperative PTH levels |
| --- | --- | --- | --- | --- |
| Selected patients | 49.7±8.44 | 20/14 | 84 (60-144) | 852.0 (637.5-1058.78) |
| Unselected patients | 47.6±11.11 | 378/300 | 84(60-108) | 1353.8  (896.4-2087.0) |
| P value | P>0.05 | P>0.05 | P>0.05 | P<0.001 |

**Supplemental Table 1. The baseline characteristics of the study participants and those excluded.**
