# Supplementary material for: Changes in bone mineral density after total parathyroidectomy without autotransplantation in the end-stage renal disease patients with secondary hyperparathyroidism
Source: BMC Nephrol. 2018 Jun 15;19:142. doi: 10.1186/s12882-018-0934-1 (PMC6003160; doi:10.1186/s12882-018-0934-1)
Supplement: Supplementary file 2 — Table S2. Results of dual X-ray absorbtiometry (DXA) before and after total parathyroidectomy (PTX). (DOC 51 kb) [file 12882_2018_934_MOESM2_ESM.doc]

**Supplemental Table 2. Results of dual X-ray absorbtiometry (DXA) before and after total parathyroidectomy (PTX)**

|  | | | **BMD (g/cm2)** | **T score** | **Osteoporosis** | **Osteopenia** |
| --- | --- | --- | --- | --- | --- | --- |
| **lateral spine** | **L1** | Pre-PTX | 0.91±0.158 | -1.11±1.278 | 4(11.8%) | 14(41.2%) |
|  |  | Post-PTX | 1.01±0.188* | -0.23±1.562** | 2(5.9%) | 9(26.5%) |
|  | **L2** | Pre-PTX | 0.94±0.155 | -1.14±1.284 | 6(17.6%) | 13(38.2%) |
|  |  | Post-PTX | 1.06±0.181** | -0.05±1.558** | 1(2.9%) | 8(23.5%) |
|  | **L3** | Pre-PTX | 0.94±0.158 | -1.42±1.381 | 8(23.5%) | 13(38.2%) |
|  |  | Post-PTX | 1.07±0.173** | -0.18±1.535** | 1(2.9%) | 10(29.4%) |
|  | **L4** | Pre-PTX | 0.89±0.173 | -1.72±1.514 | 11(32.4%) | 11(32.4%) |
|  |  | Post-PTX | 1.03±0.176** | -0.39±1.552** | 2(5.9%) | 11(32.4%) |
|  | **Total** | Pre-PTX | 0.92±0.157 | -1.39±1.341 | 6(17.6%) | 16(47.1%) |
|  |  | Post-PTX | 1.04±0.171** | -0.25±1.485** | 2(5.9%) | 10(29.4%) |
| **Hip** | **Neck** | Pre-PTX | 0.69±0.127 | -1.68±0.942 | 8(23.5%) | 19(55.9%) |
|  |  | Post-PTX | 0.79±0.131** | -0.84±1.009** | 0(0%) | 19(55.9%) |
|  | **Troch** | Pre-PTX | 0.58±0.114 | -1.44±0.949 | 2(5.9%) | 21(61.8%) |
|  |  | Post-PTX | 0.64±0.106** | -0.88±0.869** | 0(0%) | 21(61.8%) |
|  | **Internal** | Pre-PTX | 0.92±0.210 | -1.37±1.175 | 6(17.6%) | 17(50.0%) |
|  |  | Post-PTX | 1.02±0.182* | -0.79±1.014* | 1(2.9%) | 15(44.1%) |
|  | **Total** | Pre-PTX | 0.79±0.167 | -1.47±1.145 | 6(17.6%) | 18(52.9%) |
|  |  | Post-PTX | 0.88±0.145* | -0.84±0.988* | 1(2.9%) | 18(52.9%) |

Data are expressed as mean ± S.D. * p<0.05 vs. preoperatively; ** p<0.001 vs. preoperatively;
