# Supplementary figures and images for: Changes in bone mineral density after total parathyroidectomy without autotransplantation in the end-stage renal disease patients with secondary hyperparathyroidism
Source: BMC Nephrol. 2018 Jun 15;19:142. doi: 10.1186/s12882-018-0934-1 (PMC6003160; doi:10.1186/s12882-018-0934-1)

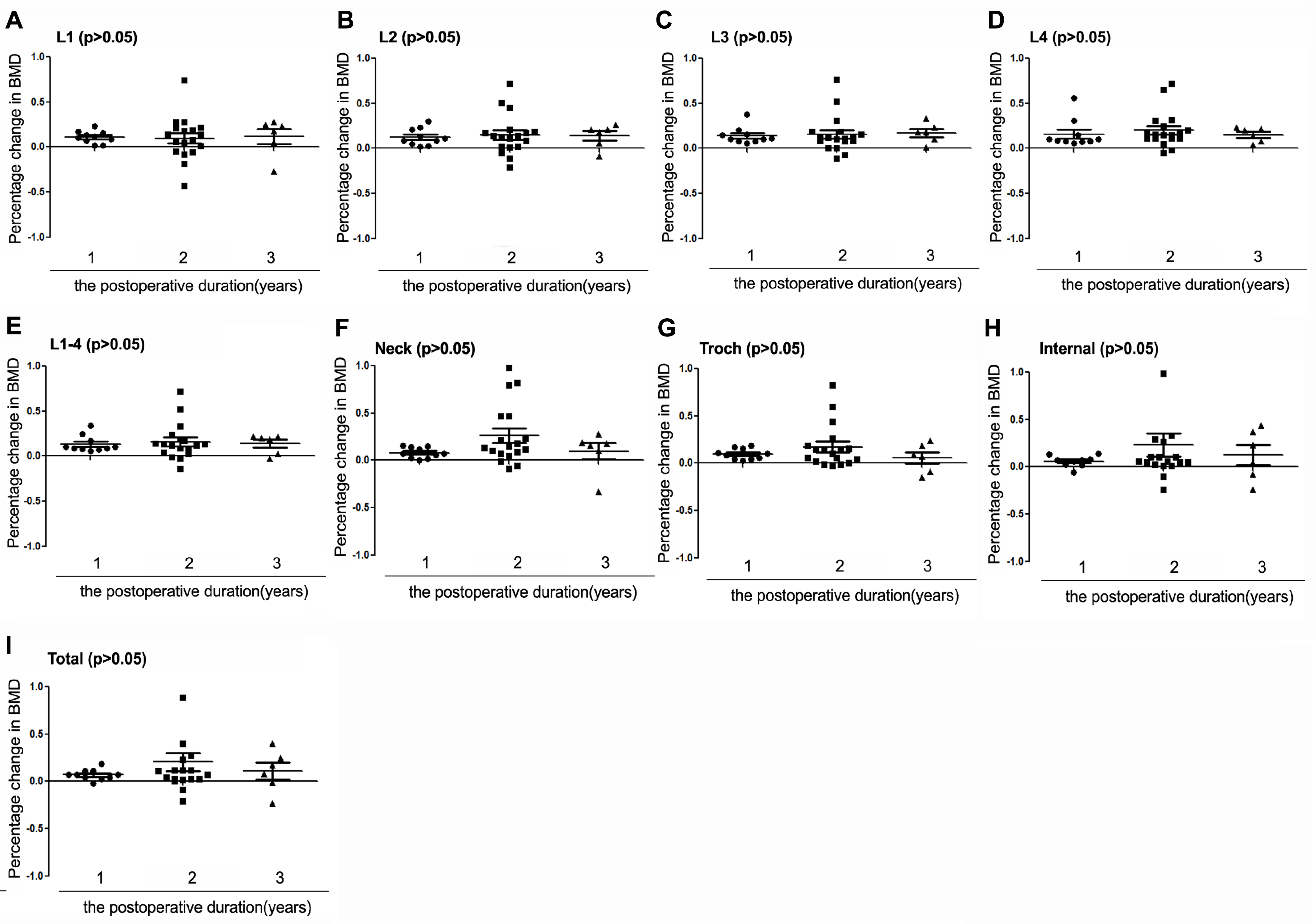

Supplement: Supplementary file 3 — Figure S1. BMD changes after surgery in the participants with different postoperative durations. A-E: BMD changes in the lumbar spine region; G-I: BMD changes in the hip region. (TIF 37610 kb) [file 12882_2018_934_MOESM3_ESM.tif]
